# Intermittent antibiotic exposure of *Escherichia coli* biofilms drives resistance in catheter-associated infection models

**DOI:** 10.1038/s41522-025-00906-4

**Published:** 2026-01-17

**Authors:** Yutaka Yoshii, Stanislas Thiriet-Rupert, David Lebeaux, Jean-Marc Ghigo, Christophe Beloin

**Affiliations:** 1 Institut Pasteur, Université Paris Cité, Genetics of Biofilms Laboratory, CNRS UMR6047, Paris, France; 2 Department of Infectious Diseases, Saint Louis-Lariboisière Hospital, Assistance Publique-Hôpitaux de Paris, Université Paris Cité, Paris, France; 3 Present Address: Université Paris Cité, Université Sorbonne Paris Nord, Inserm, IAME, Paris, France

**Keywords:** Diseases, Microbiology

## Abstract

The use of antibiotic lock therapy (ALT) to protect catheters from infection is still being debated due to its inconsistent effectiveness and the potential risk of promoting antibiotic resistance. Using an in vitro infection model of a pediatric venous access port, we demonstrated that 10 days of continuous therapy eradicates *Escherichia coli* biofilms in vitro without the emergence of antibiotic resistance. By contrast, an 8-h intermittent therapy used for infected parenteral nutrition patients rapidly selected low-level amikacin-resistant mutants both in vitro and in vivo in a clinically relevant rat model, primarily due to convergent *fusA*, *sbmA*, and *cpxA* mutations. Our findings indicate that intermittent dosing generates pulsed selective pressure, favoring the development of resistance mutants within spatially structured biofilm communities. This suggests that biofilms may act as evolutionary incubators, in which medical interventions could unintentionally influence adaptation outcomes. Furthermore, the low-level resistance developing in treated biofilms may be overlooked in clinical settings and contribute to the selection of high-level resistant mutants. Our study, therefore, underscores that, in addition to dosing, optimizing the timing of antimicrobial treatment could mitigate the emergence of resistance. These principles are applicable beyond catheters to any biofilm-related infections where short-term antibiotic exposure may impact microbial community adaptation.

## Introduction

Antimicrobial resistance (AMR) is rapidly emerging in a group of bacterial species collectively known by the acronym ESKAPEE (*Enterococcus faecium*, *Staphylococcus aureus*, *Klebsiella pneumoniae*, *Acinetobacter baumannii*, *Pseudomonas aeruginosa*, *Enterobacter* species, and *Escherichia coli*), posing a global threat to human and animal health^1,2^. Among the ESKAPEE group, the Gram-negative bacterium *E. coli* is a leading cause of AMR-related morbidity and mortality worldwide^1^. Whereas *E. coli* is generally susceptible to many antimicrobial agents, the misuse of antibiotics or ill-adapted treatment regimens can rapidly lead to resistance^3,4^. Considering the difficulties in developing new antibiotics, understanding how *E. coli* evolves antibiotic resistance during and after antibiotic treatment could mitigate the impact of AMR in this bacterium^5^.

Pathogenic *E. coli* are well-known agents of acute gastrointestinal and chronic urinary tract infections, but also responsible for recalcitrant catheter infections^6–10^. Among the causative pathogens of central venous catheter-related infections, although *Staphylococcus aureus* and coagulase-negative staphylococci remain the most prevalent microorganisms^11^, recent surveillance data reveal a growing proportion of gram-negative bacteria, including *E. coli*, which account for 9.7–20% of these infections^6,11–15^. Therefore, in clinical settings, the significance of *E. coli* as a cause of catheter-related infections, exhibiting high and multifactorial antibiotic tolerance due to biofilms^16,17^, has gained increased attention^18^.

Laboratory evolution experiments on biofilms under antibiotic treatments with sub- to above-minimum Inhibitory concentration (MICs) for short to long periods showed that biofilm tolerance could serve as a stepping stone for AMR^19–25^. Using an in vitro biofilm model on silicone disks, we recently demonstrated that *E. coli* biofilms evolved resistance faster and at higher levels than planktonic cells after repeated treatments with the aminoglycoside amikacin at above-MIC concentrations^26^. However, the evolutionary dynamics of biofilms are highly influenced by environmental and experimental factors, such as bacterial strains, inoculum sizes, nutrient levels, and experimental duration^27^. Understanding the emergence of AMR in biofilms exposed to clinically relevant conditions is therefore crucial for developing effective treatments and limiting antibiotic resistance.

In this study, we used in vitro and in vivo preclinical rat models to assess the efficacy and risks of the emergence of antibiotic resistance after continuous and intermittent treatment for totally implantable venous access ports (TIVAP), a medical device widely used in clinics to administer intravenous drips or nutrients^28,29^. These TIVAPs, which are composed of a subcutaneous injectable chamber connected to the venous circulation by a catheter, are routinely contaminated upon manipulation or anterograde colonization, often leading to biofilm infections^29,30^. The standard treatment to salvage or protect these devices consists of an antibiotic lock therapy (ALT), during which high concentrations of antibiotics, typically 100–1000 times the MIC, are injected into the catheter lumen. This treatment is performed continuously for 7–14 days or intermittently in parenteral nutrition infusion and hemodialysis cases^29,31–35^, with mixed clinical efficiency outcomes^36–38^. Moreover, concerns are rising about antibiotic resistance observed after intermittent ALT in hemodialysis patients^39,40^.

Here, we investigated the efficacy and AMR-associated risks of continuous and intermittent ALT amikacin (ALT_AMK_) on biofilm in pediatric TIVAP, both in vitro and in vivo. We showed that a 10-day continuous ALT_AMK_ efficiently eradicated *E. coli* biofilms without favoring the emergence of resistance. In contrast, intermittent ALT_AMK_ not only failed to fully eradicate *E. coli* biofilms but also promoted the emergence of antibiotic resistance to amikacin. This study highlights the need to consider potential evolutionary paths toward antibiotic resistance in biofilm-associated infections and provides valuable insights for developing a safer and more efficient management of catheters.

## Results

### Continuous 10- and 7-day ALT treatments of *E. coli* biofilms with amikacin do not promote the emergence of antibiotic resistance

**Fig. 1:**
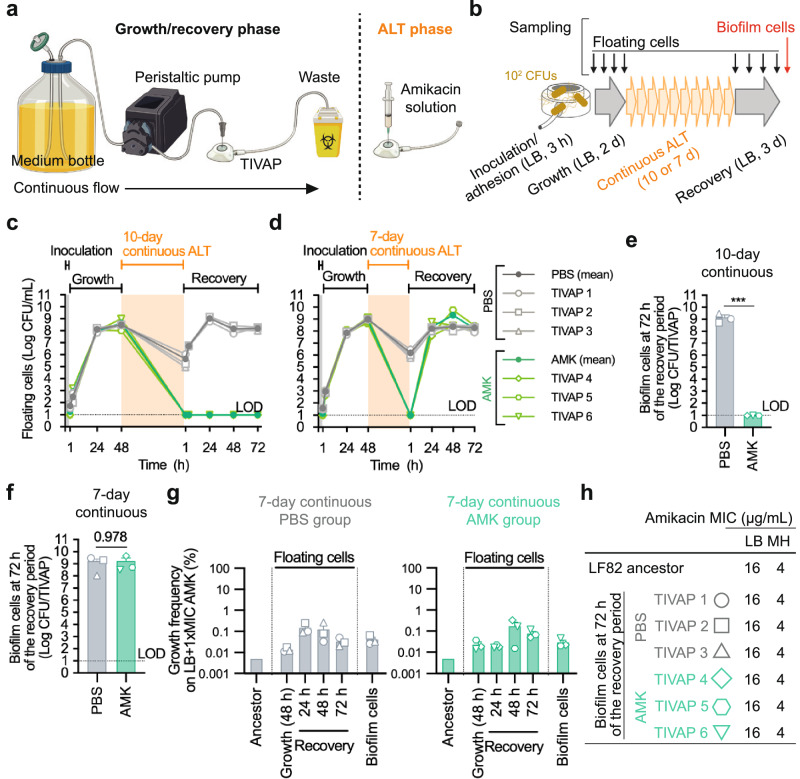
10- and 7-day continuous amikacin lock therapy at 500 times the MIC for *E. coli* LF82 biofilms formed in vitro on TIVAP under continuous LB flow. **a** Schematic illustrations of the in vitro TIVAP model, including growth/recovery and antibiotic lock therapy (ALT) phases. **b** Experimental protocol for 10- and 7-day daily-exchanged continuous amikacin lock therapy (ALT_AMK_) at 500 times the MIC. Experiments were conducted with 3-h bacterial inoculation/adhesion on TIVAPs without continuous flow, followed by 2-day biofilm growth under continuous LB flow, 10- or 7-day ALT without continuous flow, and 3-day biofilm formation recovery with continuous LB flow. PBS was injected into TIVAPs as the control (ALT_PBS_). **c**, **d** Time course of CFUs of floating cell samples collected from TIVAP catheter tips in the 10- (**c**) or 7-day (**d**) experiments. **e**, **f** CFUs of biofilm samples at the endpoint in the 10-(**e**) and 7-day (**f**) experiments. **g** Growth frequencies of floating cell and biofilm samples in the 7-day experiment on amikacin plates with one time the MIC (ALT_PBS_ control group on the left panel and ALT_AMK_ group on the right panel). **h** Amikacin MIC for biofilm population samples at the endpoint on LB and Mueller-Hinton (MH) media. The tested biofilm samples were the same as (**f**). The data used in (**c**, **e**) were extracted from the same 10-day experiment, and the data in (**e**–**g**) were extracted from the same 7-day experiment. The limit of detection (LOD) indicates the lowest CFU that can be measured. Values below the LOD were included and plotted at the LOD threshold. Means, individual values, and standard errors from three biological replicates (TIVAPs) are shown (Welch’s *t*-test, ***p < 0.001). Figure 1a was generated using BioRender.

To study the potential emergence of AMR in biofilms in clinical devices, we established an in vitro model using pediatric TIVAP, where biofilms of the adherent-invasive *E. coli* strain LF82^41^ composed of ca 4.0 × 10^8^ cells/TIVAP were formed after 48-h continuous flow of LB bacterial growth medium (Fig. 1a and Supplementary Fig. 1a–d). This model was subjected to amikacin antibiotic lock therapy (ALT_AMK_), demonstrating that 48-h *E. coli* LF82 biofilms are sensitive to a 1-day ALT_AMK_ in a dose-dependent manner (Supplementary Fig. 1e, f). The ALT_AMK_ at a concentration of 500 times the MIC resulted in approximately a five-log reduction in free-floating and biofilm cells compared to the PBS control (ALT_PBS_), confirming that the ALT_AMK_ effectively reduces the survival of biofilm cells in our in vitro TIVAP system.

Considering that guidelines for the treatment of TIVAP infections recommend consecutive 7–14 days of ALT using antibiotic concentrations at 100–1000 times the MIC^32–35^, we compared the effects of a daily-exchanged ALT_AMK_ at 500 times the MIC for 10 and 7 days (Fig. 1b–f). Ten-day continuous ALT_AMK_ treatment prevented bacterial regrowth and successfully eradicated biofilm bacteria at the endpoint (Fig. 1c, e). By contrast, despite showing undetectable levels of free-floating cells 1 h after the recovery phase, the 7-day treatment allowed bacterial regrowth after the 3-day recovery phase in all three tested TIVAPs (Fig. 1d and Supplementary Fig. 2a). The final bacterial counts in the 7-day ALT_AMK_ group were almost equivalent to the ALT_PBS_ control group, with ~10^8^ colony-forming units (CFU)/mL of free-floating cells and 10^9^ CFU/TIVAP in biofilm samples (Fig. 1d, f).

We also assessed the development of amikacin resistance in the recovered bacterial population during the 7-day continuous ALT_AMK_ experiment by measuring growth frequency on amikacin plates with one time the MIC. The ALT_AMK_-treated biofilm populations showed growth frequencies of less than 0.1%, like those of the ALT_PBS_ control group (Fig. 1g and Supplementary Fig. 2b). Additionally, the population amikacin MICs for treated and control biofilm bacteria were 16 μg/mL in LB and 4 µg/mL in Mueller-Hinton (MH), matching those of the ancestor (Fig. 1h and Supplementary Fig. 2c). Consistent with this absence of increase in MICs, no genetic mutation in genes related to aminoglycoside antibiotic resistance was identified in endpoint biofilm populations from these ALT_PBS_ and ALT_AMK_ groups (Supplementary Data 1). This demonstrated that the 7-day continuous ALT_AMK_, while inefficient regarding biofilm eradication, did not cause the emergence of amikacin resistance.

### Intermittent ALT treatment of TIVAP in vitro biofilms leads to low-level amikacin resistance

**Fig. 2:**
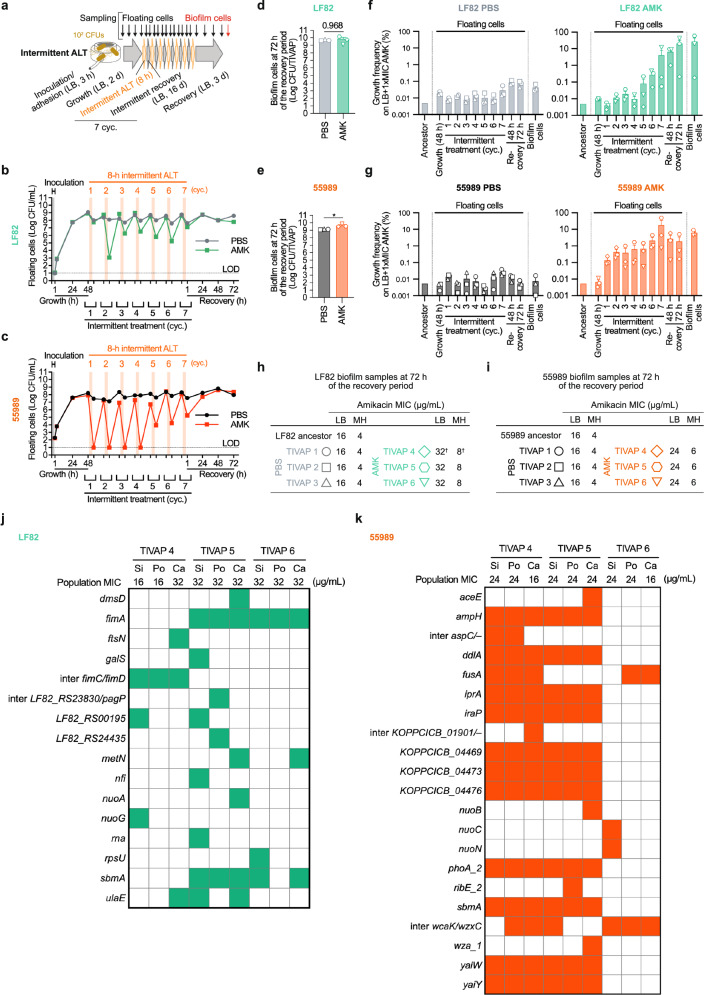
Seven cycles of intermittent amikacin lock therapy at 500 times the MIC for in vitro *E. coli* LF82 and 55989 TIVAP biofilms under continuous LB flow. **a** Experimental protocol: Seven cycles of 8-h intermittent amikacin lock therapy at 500-fold MIC (ALT_AMK_) were conducted on biofilms formed by *E. coli* LF82 and 55989 in TIVAPs. The control group received PBS (ALT_PBS_). The experiment included 3-h bacterial inoculation and adhesion without continuous flow, followed by 2 days of biofilm growth under continuous LB flow, and subsequent alternating 8-h ALT and 16-h recovery phases, culminating in a 3-day biofilm recovery with continuous flow. **b**, **c** CFU time course: Time course data of CFUs from floating cell samples collected from the catheter tips of TIVAPs exhibiting LF82 (**b**) and 55989 (**c**) biofilms were analyzed. Both panels compared the floating cell dynamics in response to ALT_AMK_ and ALT_PBS_. **d**, **e** Endpoint biofilm CFU: The CFUs at the endpoint for LF82 (**d**) and 55989 (**e**) biofilm samples on TIVAPs were measured. **f**, **g** Growth frequencies on amikacin plates: Growth frequencies of LF82 (**f**) and 55989 (**g**) floating cell and biofilm samples on amikacin plates with one time the MIC were measured (ALT_PBS_ group on the left panel and ALT_AMK_ on the right panel in each). **h**, **i** Amikacin MIC determination for biofilm populations: The amikacin MIC values for LF82 (**h**) and 55989 (**i**) biofilm populations were assessed on LB and MH media. **j**, **k** Whole-population genome sequencing (WGS)-based mutational analysis: Heatmaps identifying mutations from endpoint biofilm populations, determined by WGS, are displayed in panels **j** for LF82 and **k** for 55989. Of note, mutations at frequencies of ≥20% for LF82 and ≥10% for 55989 are shown (see Supplementary Data 1 for all identified mutations). Si, Po, and Ca represent biofilm samples from the silicone septum, port, and catheter parts of each TIVAP, respectively. The data used in (**b**, **d**, **f**, **h**, **j**) were extracted from the same LF82 intermittent amikacin lock experiment, and the data in (**c**, **e**, **g**, **i**, **k**) were extracted from the same 55989 intermittent amikacin lock experiment. The limit of detection (LOD), which indicates the lowest CFU that can be measured, was established as 10 CFU/mL. Values below LOD were included and plotted at the LOD threshold. Means, individual values, and standard errors from three biological replicates (TIVAPs) are shown (Welch’s *t*-test, *p < 0.05). For †, 32 and 8 µg/mL in LB and MH were obtained from the catheter biofilm and were demonstrated as the highest amikacin MIC value compared to silicone and port biofilms (see Supplementary Fig. 3d).

We then investigated the impact of intermittent ALT_AMK_ treatment, which is recommended for patients on total parenteral nutrition (TPN) with infected long-term catheters. For TPN catheter infection treatment in clinics, antibiotics at 100–1000 times the MIC are installed in the catheter lumen for 8 to 12 h and alternated with TPN administration during the rest of the day, and treatment/nutrition cycles are repeated over several days^35^, which led to mixed clinical efficiency outcomes in some parenteral nutrition infusion and hemodialysis cases^36–38^. We assessed treatment efficacy and the emergence of amikacin resistance in two biofilm-forming *E. coli* strains, LF82 and the enteroaggregative 55989^42^. We conducted seven cycles of a 1-day cycle: 8 h of intermittent ALT_AMK_ at 500 times the MIC and 16 h of continuous LB flow without antibiotics. These cycles concluded with a 3-day recovery phase (Fig. 2a). The treatment regimen used in our study closely mimics the one used in clinics.

When 48-h biofilms were subjected to the intermittent ALT_AMK_, both strains exhibited a reduction in survival during each ALT cycle. However, this reduction diminished during the final cycle. Moreover, after the 3-day recovery phase, the numbers of free-floating and biofilm cells were similar between the ALT_AMK_ and ALT_PBS_ control groups, indicating treatment failure (Fig. 2b–e and Supplementary Fig. 3a, b, e, f).

Growth frequencies on amikacin plates at one time the MIC revealed a gradual increase in the number of free-floating cells of the ALT_AMK_ groups over the cycles. By the end of the cycles, two out of three LF82 replicates reached 10–100% growth frequencies, and all three 55989 replicates reached 1–10%. In contrast, the ALT_PBS_ control groups maintained low growth frequencies of 0.001–0.1% for both free-floating and biofilm cells of both strains (Fig. 2f, g and Supplementary Fig. 3c, g). In the ALT_AMK_ groups, the population amikacin MICs increased to 32 μg/mL in LB (8 µg/mL in MH) for all three LF82 replicates (Fig. 2h and Supplementary Fig. 3d) and to 24 µg/mL in LB (6 µg/mL in MH) for all three 55989 replicates, higher than the ancestor strains’ MICs (Fig. 2i and Supplementary Fig. 3h). According to CLSI guidelines, the MIC breakpoints for amikacin against *E. coli* in MH medium are ≤4 µg/mL for susceptible isolates and ≥16 µg/mL for resistant ones^43^. Therefore, the observed MICs were indicative of the selection of low-level antibiotic resistance.

To identify the genetic factors associated with the increased MIC and growth frequency observed in the ALT_AMK_ group, we sequenced endpoint LF82 and 55989 biofilm populations obtained from each part of ALT_AMK_-treated TIVAPs (silicone septum, port, and catheter parts of TIVAPs n°4–6). Mutations with a frequency of 20% or more for LF82 and 10% or more for 55989, compared to the ancestral population, are shown in Fig. 2j, k. We identified convergence in the mutation profiles for the two strains with mutations in the *sbmA* and *fusA* genes (Supplementary Data 1). *sbmA* encodes an inner-membrane peptide transporter while *fusA* encodes the essential elongation factor G. Loss-of-function mutations in *sbmA* and mutations in *fusA* have been previously associated with increased aminoglycoside resistance^26,44–49^. The *sbmA* gene was mutated in two out of the three LF82 biofilm populations and deleted in two out of three 55989 biofilm populations, along with surrounding neighboring genes, including *phoA_2*, *iraP*, *ddlA*, *KOPPCICB_04469*, *yaiY*, *yaiW*, *KOPPCICB_04473*, *ampH*, *iprA*, and *KOPPCICB_04476* (Fig. 2j, k, Table 1, and Supplementary Data 1). The *fusA* gene was mutated in two out of three 55989 biofilm populations (Fig. 2k). Additionally, mutations in the *fusA* gene were also detected at low frequencies in two out of three LF82 biofilm populations (Supplementary Data 1). To validate that these mutations are specific to AMK treatment, we sequenced untreated PBS biofilm populations from one of the two strains, LF82. *sbmA* and *fusA* mutations were absent in this PBS control group (Supplementary Data 1). Interestingly, however, in this LF82 PBS control group, we detected mutations in the *fimA* gene, which encodes the major subunit of type 1 fimbriae. These mutations were also present in the AMK intermittently treated biofilm LF82 and 55989 populations (Supplementary Data 1). Since these *fimA* mutations were also present in the 7-day continuous control and treated LF82 biofilms, it is likely that they have been selected by the biofilm lifestyle and may affect biofilm-forming capacity in TIVAPs, hence possibly tolerance to AMK.

**Table 1:** Identified non-synonymous *fusA*, *sbmA*, and *cpxA* mutations from biofilm populations at a frequency higher than 5% in all experiments

Gene	Strain	Experiment	Sample (TIVAP)		Mutation	
***fusA***	LF82	In vitro LB	4	si	K653N	G->T
			5	ca	K653N	G->T
	55989	In vitro LB	4	si	F605L	T->C
				po	F605L	T->C
				ca	F605L	T->C
			6	po	A678V	C->T
				ca	A678V	C->T
	55989pAT881	In vitro TPN	5	si	S672F	C->T
				po	S672F	C->T
			6	si	A678V	C->T
					S672F	C->T
				po	R671H	G->A
				ca	R671H	G->A
					S672F	C->T
					E100K	G->A
	55989pAT881	In vivo	4	si	R671H	G->A
					S672F	C->T
				po	S672F	C->T
					R671H	G->A
				ca	A592V	C->T
			5	si	S672F	C->T
					R671H	G->A
				ca	S672F	C->T
					R671H	G->A
					R671C	C->T
			6	ca	S672F	C->T
					R671H	G->A
***sbmA***	LF82	In vitro LB	5	si	W27*	G->A
					Q120H	A->C
				po	W27*	G->A
				ca	coding	12/1221 nt
					W27*	G->A
			6	si	C67*	C->A
				po	C67*	C->A
				ca	C67*	C->A
	55989	In vitro LB	4	si	del.	∆11024 nt^†^
				po	del.	∆11024 nt^†^
				ca	del.	∆11024 nt^†^
			5	si	del.	∆11024 nt^†^
				po	del.	∆11024 nt^†^
				ca	del.	∆11024 nt^†^
***cpxA***	LF82	In vitro LB	4	si	L19R	T->G
			5	si	L19R	T->G
				ca	L19R	T->G
			6	si	M22L	A->T
	55989pAT881	In vitro TPN	5	si	T6I	C->T
				po	T6I	C->T
				ca	T6I	C->T
			6	si	D162E	C->A
				po	D162E	C->A
				ca	D162E	C->A

Si, po, and ca represent biofilm samples from the silicone septum, port, and catheter parts of each TIVAP, respectively. Del. represents deletion of the gene. * indicates a stop codon resulting from the mutation, leading to premature termination of protein translation. †The *sbmA* gene was deleted together with neighbor genes of *phoA_2*, *iraP*, *ddlA*, *KOPPCICB_04469*, *yaiY*, *yaiW*, *KOPPCICB_04473*, *ampH*, *iprA*, and *KOPPCICB_04476*.

Overall, these results demonstrate that in vitro intermittent ALT_AMK_ treatment of TIVAP failed to eradicate biofilms and promoted the emergence of low-level resistance, potentially caused notably by mutations in *sbmA* and *fusA*.

### Emergence of resistance occurs when using a clinically relevant total parenteral nutrition medium

**Fig. 3:**
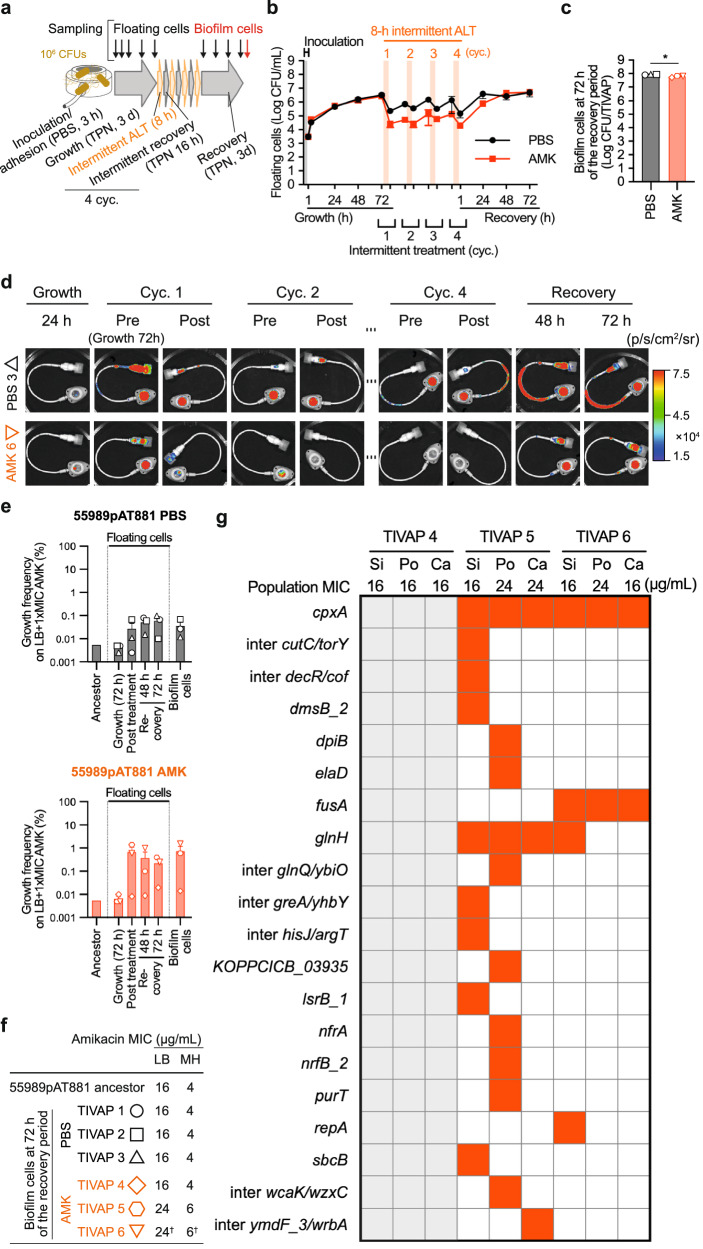
Four cycles of intermittent amikacin lock therapy at 500 times the MIC for bioluminescent 55989pAT881 in vitro TIVAP biofilms under continuous TPN flow. **a** Experimental protocol for four cycles of 8-h intermittent amikacin lock therapy (ALT_AMK_). PBS was injected into TIVAPs as the control (ALT_PBS_). Experiments were conducted with 3-h inoculation/adhesion of the PBS-suspended bacteria on TIVAPs without continuous flow, followed by 3-day biofilm growth under continuous total parenteral nutrition (TPN) flow, four-cycle sets of 8-h ALT phase (without flow) and 16-h intermittent recovery phase (with continuous TPN flow), and 3-day biofilm formation recovery with continuous TPN flow. **b** Time course of CFUs of floating cell samples collected from the catheter tips of TIVAPs, where 55989pAT881 biofilms were formed and treated with four cycles of 8-h intermittent ALT_AMK_ at 500-fold MIC or ALT_PBS_. **c** CFUs of bioluminescent 55989pAT881 biofilm samples at the endpoint. **d** Representative time course of bioluminescence images of 55989pAT881 biofilms formed on TIVAP treated with or without amikacin (ALT_PBS_ 3 and ALT_AMK_ 6). **e** Growth frequencies of 55989pAT881 floating cell and biofilm samples on amikacin plates at one time MIC (ALT_PBS_ control group on the upper panel and ALT_AMK_ group on the lower panel). **f** Amikacin MIC for 55989pAT881 biofilm population samples on LB and MH media. **g** Heatmap for identified mutations from endpoint biofilm populations by whole-population genome sequencing (WGS). Endpoint 55989pAT881 biofilm populations obtained from TIVAPs 5 and 6, and grown on amikacin plates at one time the MIC, were subjected to WGS. Mutations at a frequency greater than or equal to 10% are highlighted in orange (see Supplementary Data 1 for all identified mutations). Si, Po, and Ca represent biofilm samples from the silicone septum, port, and catheter parts of each TIVAP. Means, individual values, and standard errors from three biological replicates (TIVAPs) are shown (Welch’s t-test, *p < 0.05). For †, 24 and 6 µg/mL in LB and MH were obtained from the port biofilm and were demonstrated as the highest MICs compared to silicone and catheter biofilms.

To further explore the clinical relevance of the observed emergence of amikacin resistance in intermittent ALT_AMK_ under flow conditions, considering that total TPN in clinical settings is composed of highly controlled compounds capable of bacterial growth but not optimal compared to LB^50,51^, we tested whether using total TPN medium instead of LB in our in vitro model of TIVAP infection would influence the effectiveness of ALT_AMK_ treatment. First, we verified that the bioluminescent *E. coli* 55989 carrying the pAT881 plasmid^52^ formed biofilms under continuous TPN flow (Supplementary Fig. 4a, b). We then applied four one-day cycles of an 8-h lock/16-h regrowth regimen of intermittent ALT_AMK_ at 500 times the MIC, using TPN instead of LB at all stages of the experiment after the inoculation/adhesion step (Fig. 3a). In the ALT_AMK_ group, CFU counts of free-floating cells exhibited less survival reduction compared to what we observed in LB (Fig. 3b and Supplementary Fig. 4d), consistent with reduced bacterial load in TPN vs LB. After the 3-day recovery phase, the biofilm cells in the ALT_AMK_ group were almost equivalent to those in the control ALT_PBS_ group, indicating treatment failure (Fig. 3c and Supplementary Fig. 4f). Consistent with the CFU counts of free-floating samples over time, bioluminescence imaging revealed that biofilm bioluminescent intensities decreased during ALT_AMK_ treatment but rebounded during the recovery phase (Fig. 3d and Supplementary Fig. 4c, e). Although growth frequencies on amikacin plates at one time the MIC could not be analyzed overtime for the ALT_AMK_ group due to low bacterial counts in the floating cell samples, this group showed *ca*. tenfold increased of such frequencies (0.1–1%) in recovery-phase free-floating and endpoint biofilm samples in two out of three ALT_AMK_ group replicates (TIVAPs n°5 and 6), compared to the PBS group (0.01–0.1%). In the PBS group, resistance frequencies also increased, to a lesser extent. This latter result is consistent with the well-documented enhanced mutation rate in biofilms, increasing the likelihood of accumulating emergent antibiotic resistance mutations, even in the absence of antibiotic pressure^26,53–55^ (Fig. 3e and Supplementary Fig. 4g). The two ALT_AMK_ group replicates demonstrated low-level resistance at 24 µg/mL in LB (6 µg/mL in MH) of amikacin (Fig. 3f and Supplementary Fig. 4h).

We then sequenced endpoint biofilm populations in the two TIVAPs with the increased MIC (ALT_AMK_ TIVAPs n°5 and 6), using collected samples from amikacin plates at one time the MIC. Consistent with in vitro TIVAP experiments performed with LB, we identified convergent mutations in the *fusA* and *cpxA* genes. *fusA* mutations were detected at frequencies ≥10% in all parts of TIVAP n°6 (Fig. 3g) and at lower frequencies in TIVAP 5 (Supplementary Data 1). *cpxA* mutations were detected at frequencies ≥10% in all parts of both TIVAPs n°5 and n°6. CpxA serves as the sensor kinase of the CpxAR two-component regulatory system. Mutations in the *cpxA* gene can confer aminoglycoside resistance by regulating the *acrD* gene, encoding AcrD transporter belonging to the resistance-nodulation-division family and a component of the AcrAD-TolC efflux pump^56^.

These results confirmed that in vitro amikacin intermittent treatment of biofilms using a clinically relevant parenteral nutrition medium and regimen also fails to eradicate biofilms, leading to the emergence of low-level antibiotic resistance.

### Intermittently treated biofilm with amikacin leads to the emergence of resistance in a preclinical catheter-associated infection model

**Fig. 4:**
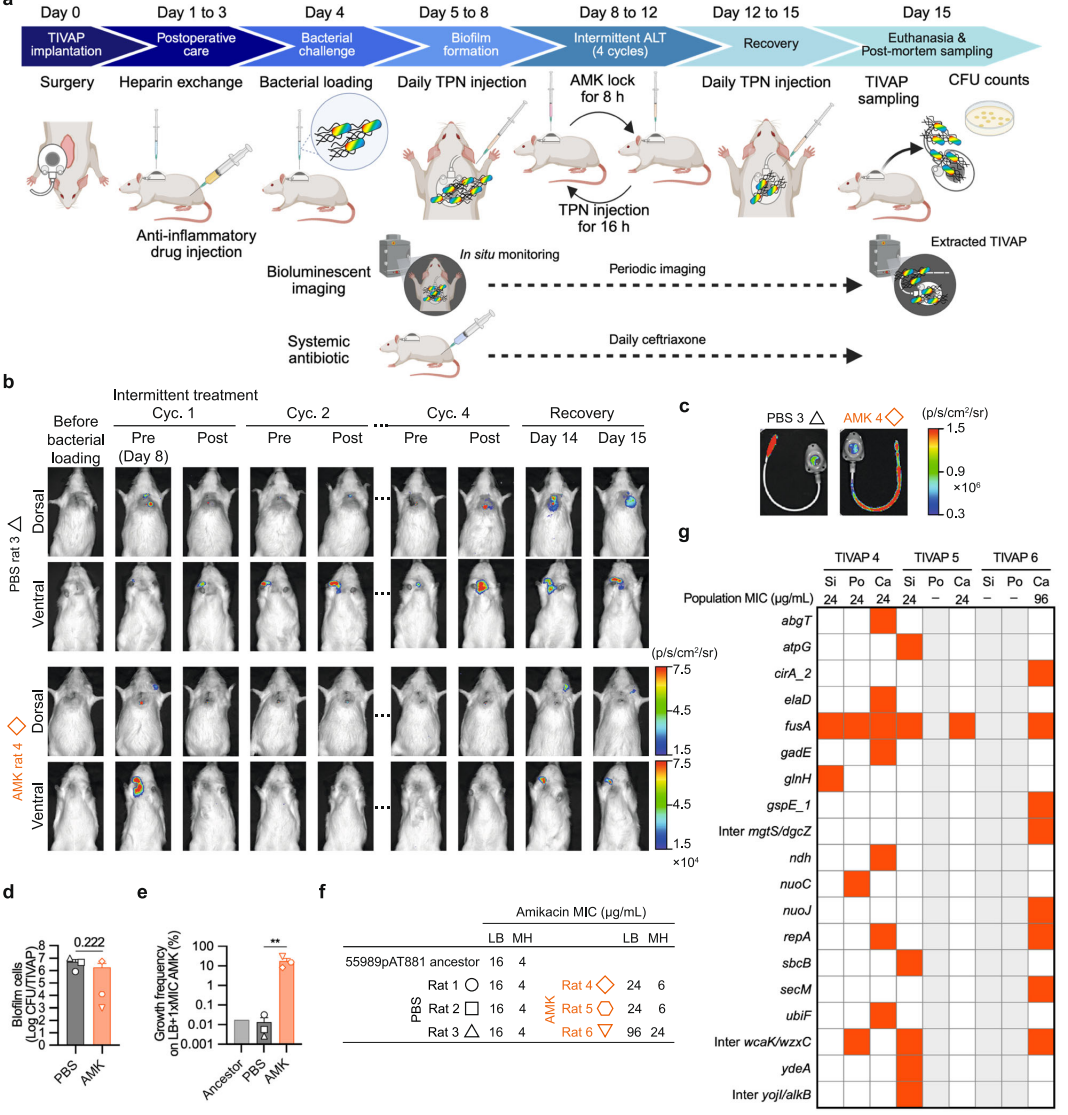
Four cycles of intermittent amikacin lock at 500 times the MIC for bioluminescent 55989 in vivo TIVAP biofilms. **a** Experimental protocol: Four cycles of 8-h intermittent amikacin lock therapy (ALT_AMK_) at 500-fold MIC were performed on biofilms formed by the bioluminescent 55989 strain in TIVAPs, with a control group receiving PBS (ALT_PBS_). Medical-grade 5 Fr TIVAPs were implanted in rats after a 1-week acclimatization. On day 4, the bioluminescent strain was inoculated into the TIVAP port. Biofilm formation was established over 3 days with daily TPN on days 5–8. The 8-h intermittent ALT and 16-h recovery phases were conducted from days 8–12, followed by a biofilm recovery phase with TPN on days 12–15. TIVAPs were collected from euthanized rats on day 15 for analysis. **b** Bioluminescence time course: Examples of bioluminescent imaging of biofilms treated with ALT_AMK_ versus ALT_PBS_ are presented (ALT_PBS_ rat 3 and ALT_AMK_ rat 4). **c** Endpoint TIVAP bioluminescence images: Extracted TIVAP images illustrating biofilm presence in each treatment group are shown. **d** Endpoint biofilm CFU: CFUs for biofilm samples were measured at the endpoint. **e** Growth frequencies on amikacin plates: Growth frequencies of biofilm cell samples on amikacin plates at 1 time the MIC are reported. **f** Amikacin MIC determination: The amikacin MIC values for biofilm cell samples grown on LB and MH media were assessed. **g** Whole-population genome sequencing (WGS)-based mutational analysis: Heatmaps showcasing mutations from endpoint biofilm populations, identified through WGS, are presented, with mutations at frequencies of ≥10% highlighted in orange (see Supplementary Data 1). Biofilms were not recovered from the ALT_AMK_ TIVAP 5 port and TIVAP 6 silicone and port (see Supplementary Fig. 5b). Si, Po, and Ca represent biofilm samples from the silicone septum, port, and catheter parts of each TIVAP, respectively. Means, individual values, and standard errors from three biological replicates (TIVAPs) are shown (Welch’s *t*-test, **p < 0.001). Figure 4a was generated using BioRender.

To validate our in vitro findings in vivo, we used a previously described rat model that reproduces most clinically relevant situations associated with TIVAP biofilm infections^52,57^ (Fig. 4a). Following TIVAP implantation, we established a 3-day biofilm of bioluminescent 55989 strain (55989pAT881) under static conditions using TPN medium. We then administered four one-day cycles of 8-hour intermittent ALT_AMK_ at 500 times the MIC and 16-h withdrawal by installing the TPN solution, concluding with a 3-day recovery phase. Throughout this period, we monitored bioluminescent biofilms in situ (Fig. 4b, c and Supplementary Fig. 5a). In the ALT_PBS_ control group, bioluminescence intensities remained stable on either the dorsal or ventral sides of all three catheterized rats (Fig. 4b). Conversely, the ALT_AMK_ group gradually reduced bioluminescence intensity, which disappeared after the second treatment cycle. However, in one rat (ALT_AMK_ Rat 4), bioluminescence intensity resurfaced during the recovery phase. Upon extracting the TIVAPs, bioluminescent bacterial biofilms were present in all rats of the ALT_PBS_ group and in ALT_AMK_ Rat 4 (Fig. 4c). Additionally, the TIVAP of ALT_AMK_ Rat 5 also demonstrated very weak bioluminescence intensity (Supplementary Fig. 5a). CFU counting revealed that all parts of the ALT_PBS_ group and six out of nine parts in the ALT_AMK_ group recovered biofilm cells at the endpoint. As a result, the ALT_AMK_ group tended to have lower CFU numbers of biofilm bacteria on extracted TIVAPs than the ALT_PBS_ group, although the difference was not statistically significant (Fig. 4d and Supplementary Fig. 5b). This lack of significance may be due to the small size of the sample (*n* = 3 per group), which could limit the statistical power of the analyses and make it more challenging to identify true differences between the groups. Small amounts of biofilm bacteria were found on TIVAPs from ALT_AMK_ Rat 6, although bioluminescence was not detected during the recovery phase (Supplementary Fig. 5a,b). Overall, these results indicate that the in vivo intermittent ALT_AMK_ treatment failed to eradicate biofilms in all three animals in our preclinical model.

More concerning was the observed higher growth frequency of biofilm samples on amikacin plates at one time the MIC in the ALT_AMK_ group, ranging from 10–100%, compared to the ALT_PBS_ control group (Fig. 4e and Supplementary Fig. 5c), with population amikacin MICs reaching 24–96 μg/mL in LB (6 to 24 µg/mL in MH) (Fig. 4f and Supplementary Fig. 5d). Sequence analysis of surviving endpoint biofilm populations obtained from three ALT_AMK_-treated TIVAPs confirmed that, in vivo as well, the *fusA* mutation is the most common mutation observed in all six parts of the three TIVAPs (Fig. 4g).

Taken together, our experiment demonstrated that treatment of biofilms formed in a clinically relevant in vivo TIVAP model also led to the emergence of amikacin resistance.

## Discussion

This study examined the impact of high-concentration amikacin antibiotic lock therapy (ALT_AMK_) recommended by guidelines for treating catheter-related *E. coli* biofilms. Our findings reveal how treatment frequency (timing) modulates eco-evolutionary dynamics in clinically relevant biofilm ecosystems, where pulsed antibiotic exposure creates strong selection windows for resistance emergence.

We found that a continuous 10-day lock regimen successfully eradicated in vitro LF82 biofilms, whereas a 7-day regimen did not. The favorable results associated with the 10-day continuous lock, compared to the 7-day lock, were likely due to the superior total exposure and possibly accumulation of AMK within the biofilms over the extended period. These additional 3 days of AMK locking may have reduced the likelihood of any residual tolerant bacterial regrowth once the treatment was completed. Despite this, the 7-day lock regimen still prevented the rise of resistant subpopulations. Although the link between treatment duration and resistance is debated^58^, treatment failure from short-term regimens, even if not leading to the emergence of antibiotic resistance, still requires retreatment and is therefore associated with a significant health and economic burden^59^. Mathematical modeling and clinical studies support the effectiveness of longer treatment durations, recommending at least 10 days of continuous ALT for successful catheter disinfection^32,35,60,61^. Our data were consistent with these findings, underscoring the clinical relevance of our in vitro model.

In contrast to continuous treatments, all three intermittent ALT_AMK_ treatments conducted in vitro failed, leading to the emergence of amikacin resistance with frequencies ranging from 1% to over 10%. This demonstrates how intermittent dosing turns biofilms into evolutionary incubators, where spatial structuring accelerates mutant selection. Analysis of free-floating cells revealed differences in bacterial numbers and resistance rates between LF82 and 55989 biofilms in LB, despite having the same amikacin MIC value of 16 µg/mL. Previous reports demonstrated that, in addition to biofilm formation capacity, the trajectory of antibiotic resistance evolution after antibiotic exposure is strain-specific^62–64^, suggesting that the genetic differences between LF82 and 55989 might affect biofilm formation capacity and, consequently, their tolerance to amikacin. Although the reduction in medium richness (when comparing TPN with LB media) slightly decreased the frequency of resistance observed during intermittent treatments in the in vitro TPN experiment, the bacterial load on TIVAP in the experiment ranged from 10^7^ to 10^8^ CFU/TIVAPs, comparable to what is found in clinical situations where bacterial loads on devices typically range from 10^2^ to 10^7^ CFUs/device^65–67^. Previous studies show strain-specific genetic diversification in biofilm populations^68^, suggesting that high-level resistance is selectively promoted during antibiotic treatment when nutrient levels are high^69^. Thus, while nutrient heterogeneity influences evolutionary trajectories, intermittent selection pressure remains the primary driver of resistance amplification in these structured communities. While our study demonstrates that intermittent ALT induces antibiotic resistance, future studies should consider the nature of the tested bacterial strains and nutritional conditions to better mimic clinical settings.

We also showed that intermittent treatment, performed in vivo in our preclinical rat catheter biofilm model, promoted the development of amikacin resistance, consistent with our in vitro observations. Despite varying bacterial counts at the endpoint (10^3^ to 10^7^ CFU/TIVAP), these counts are comparable with those observed in the clinical studies^65–67^. Moreover, growth frequencies on amikacin plates at one time the MIC were high for all three rats treated with ALT_AMK_, around 10%, compared to the ALT_PBS_ control (0.01%). This increased resistance seemed to develop faster and at higher levels than in vitro, which is consistent with another in vivo study on ciprofloxacin resistance in *P. aeruginosa* lung infection in mice^70^. The mechanisms behind this rapid and high-level resistance are unclear. However, the host immune environment, failing to cure the infectious disease, could finally synergize with antibiotic selection to accelerate the evolution of resistance. For instance, the host’s reactive oxygen species, which can be triggered by infections, are not only bactericides that eliminate infections but also, conversely, promote the development of antibiotic resistance in bacteria^70,71^. In our in vivo experiment, since ceftriaxone is provided as a systemic treatment, it can also be considered that the temporary presence of blood containing ceftriaxone during the transition from ALT treatment to TPN infusion influences the in vivo dynamics of amikacin resistance selection. Further in vivo investigation is needed to clarify these dynamics. For instance, using a continuous drip infusion pig catheter model^72^, in which continuous nutrition infusion is more feasible than in our rat in vivo model, could provide more clinically relevant results.

In both our in vitro and in vivo intermittent ALT experiments with lethal antibiotic concentrations, endpoint biofilm populations exhibited low levels of resistance. We observed only a 1.5 to 2.0-fold increase in MICs for most conditions, rising from 4 to 6–8 µg/mL when measured in MH, and from 16 to 24–32 µg/mL when measured in LB. These MIC values remained below the CLSI-defined resistant breakpoint^43^. Meanwhile, one specific in vivo condition (ALT_AMK_ rat 6) exceptionally showed a more significant increase, with a sixfold rise from 4 to 24 µg/mL in MH and from 16 to 96 µg/mL in LB. Additionally, similar studies have shown that biofilms of other bacteria also develop antibiotic resistance when exposed to various antibiotics^20,22,24,73^. These stepwise adaptations exemplify how biofilm communities serve as reservoirs for incremental evolution under pulsed selection. In clinical contexts, such low-level resistance increases might be easily overlooked during standard routine MIC testing, leading to repeated administration of the same antibiotic, then causing the selection of high-level resistant mutants. For example, studies have shown that in vancomycin-treated *S. aureus* bloodstream infections, low-level resistant variants—present initially as minor subpopulations that routine susceptibility tests still classify as “susceptible”—can become enriched under drug pressure once treatment begins. This enrichment may contribute to treatment failure and the eventual development of higher-level resistance^74–76^. A similar pattern has also been observed in in vitro^77^ or in vivo experiments^78^. Therefore, as discussed previously^79^, these clinical and experimental data, including our findings, emphasize the importance of low-level resistance mutants as a pathway to clinically relevant high-level resistance.

Genome sequencing revealed that the convergent mutations in the *fusA*, *sbmA*, and *cpxA* genes may have contributed to the increased resistance to amikacin observed in endpoint biofilm populations. The *fusA* gene is essential for mRNA translocation in the ribosome and is conserved in diverse bacteria. A very recent study demonstrated that elongation factor G mutations selectively silence drug-bound ribosomes, thereby preventing error cluster formation and reducing the second self-promoted uptake phase of aminoglycosides into bacteria by preserving proteome and membrane integrity^49^. Our study found that *fusA* gene mutations are observed in all four experimental conditions treated with intermittent ALT_AMK_ (in vitro LB LF82, in vitro LB 55989, in vitro TPN 55989pAT881, and in vivo 55989pAT881 experiments; Table 1), at frequencies greater than or equal to 5%. Other research also found *fusA* point mutations in laboratory and clinical isolates of different bacteria, *S. aureus*, Salmonella, and *P. aeruginosa*, warranting *fusA* mutation risks across species^80–86^.

We also identified nonsense and deletion mutations in the *sbmA* gene, encoding an inner-membrane peptide transporter, from in vitro LF82 LB and 55989 LB experiments. Deleting the *sbmA* gene has been reported to reduce susceptibility to aminoglycosides, which aligns with our results^26,44–48^. An *Enterobacter cloacae* clinical isolate has exhibited aminoglycoside resistance due to the loss of the *sbmA* gene and ~20,000 nucleotides of adjacent genes^87^. This massive deletion is similar to our findings that biofilm populations of TIVAP 4 and 5 from the in vitro 55989 experiment lost 11,024 nucleotides, including the *sbmA* and neighbor genes (Table 1), suggesting that this region might be a fragile spot where breaks occur in some bacteria.

Point mutations in the *cpxA* gene, encoding CpxA histidine kinase of the CpxRA signal transduction system, were also identified from in vitro experiments with LF82 in LB (at low frequencies, less than 20%; see Supplementary Data 1) and 55989pAT881 in TPN. The CpxRA system senses cell envelope stresses, regulating the transcription of downstream genes^88^. Some point mutation mutants of the *cpxA* gene have been demonstrated to emerge from a clinical Klebsiella strain isolated from a patient after antibiotic treatment^89^ and to increase resistance to aminoglycosides^44,45,90^, highlighting their clinical significance. These single amino acid substitutions could alter the functionality of the CpxRA system, affecting various regulons, including the *acrD* gene. Similar to the previously reported point mutations, the *cpxA* mutants identified in our study are likely to be linked with amikacin resistance. Collectively, convergent evolution in stress-response pathways underscores how antibiotic pulses reshape regulatory networks in biofilm-adapted populations.

Although the amikacin MICs of biofilm populations obtained from the TIVAP 4 catheter in the in vitro LB LF82 intermittent experiment and the TIVAP 6 silicone septum in the in vitro LB 55989 experiment were also slightly elevated at 32 µg/mL and 24 µg/mL, respectively, we detected no mutations in the previously mentioned *sbmA*, *cpxA*, and *fusA* genes (Table 1). Nevertheless, mutations in the major facilitator superfamily transporter genes and the NADH-quinone oxidoreductase subunits *nuoA·C/D* genes were observed in the biofilms from LF82 TIVAP 4; mutations in the NADH-quinone oxidoreductase subunits *nuoC/D·N* genes were observed in the biofilms from 55989 TIVAP 6, which are linked to aminoglycoside resistance, tolerance, and persistence^91–94^ (Supplementary Data 1). These findings suggest that these other mutated genes may also have collectively contributed to the development of low-level resistance.

Furthermore, mutations in the *fimA* and *fimH* genes, which encode fimbrial and adhesin proteins known to influence biofilm formation^95–97^, were identified in some biofilms during continuous and intermittent in vitro LB experiments with LF82, in vitro TPN, and in vivo studies (Supplementary Data 1). The emergence of mutations in these genes may have increased biofilm formation, leading to a higher frequency of persister cells and overall population tolerance. Consequently, along with the emergence of amikacin-resistant genes, the *fimA* and *fimH* mutations are believed to contribute to enhanced biofilm survival and tolerance to the antibiotic, promoting the emergence of resistance mutations^26^.

In conclusion, this study demonstrated that an 8-h intermittent ALT regimen leads to low-level antibiotic resistance, primarily due to *fusA*, *sbmA*, and *cpxA* mutations in two biofilm-forming *E. coli* strains under various nutritional conditions, as observed in both in vitro and preclinical in vivo catheter models. These findings establish treatment frequency as a critical evolutionary driver in clinically relevant biofilm ecosystems. Additionally, intermittent treatment can also occur in other infections because of low compliance to prescribed medication regimens. Such irregular dosing can also adversely affect treatment efficacy and potentially contribute to antibiotic resistance. Hence, while mathematical models and some promising data suggest the potential of intermittent regimens for bacterial biofilm eradication^98–105^, further research is needed to optimize the dose and management schedule to enhance their effectiveness and minimize resistance development.

## Methods

### Bacterial strains and growth conditions

The bacterial strains used in this study are listed in Supplementary Table 1. Overnight cultures were grown at 37 °C at 180 rpm in Miller’s LB (Corning Inc., cat. 46-050-CM, Corning, NY, USA) or Mueller-Hinton broth (Beckton, Dickinson and Company (BD), cat. 212322, Franklin Lakes, NJ, USA), with 50 µg/mL spectinomycin (Sigma, S9007, St Louis, MO, USA) for the 55989pAT881 strain.

### In vitro TIVAP experiments using LB broth or total parenteral nutrition (TPN) infusion

#### System setting up

The in vitro TIVAP model described in the previous paper^52^ was employed, and some modifications were made in this study. Briefly, first, medical-grade pediatric 5 Fr TIVAPs (POLYSITE 2005 Micro kit, Perouse Medical, Ivry-le-Temple, France) were prepared using a 10 cm catheter and an accessory connector at the tip of the catheter, as provided with the kit (the internal volume of the TIVAP is ~250 µL). TIVAPs were connected to a medium bottle and a waste container via a 22G Huber needle (VYGON, Inject-site, cat. 1057.27, Écouen, France) and connecting tubes (TYGON flexible plastic tube S3 E-3603, Saint-Gobain Performance Plastics, Courbevoie, France). Fresh LB medium or the TPN infusion (Baxter International, PERIOLIMEL N4E, cat. 3865924, Deerfield, IL, USA) was stored in the medium bottle and supplied from the medium bottle to the waste container through the inside of the TIVAPs by a peristaltic pump (Watson MARLOW, cat. 205S, Falmouth, UK) (Fig. 1a and Supplementary Fig. 1a). Several luer plugs were used to connect each part (Fisher Scientific, ARK-PLAS, LAX22-PP0, LHX10-PP0, LNC31-PP0, LNC69-PP0, Pittsburgh, USA). All devices were sterilized before use. This system was placed in a microbiological safety cabinet to maintain sterility. The experimental temperature was maintained at 37 °C with a heater (Heidolph Incubator 1000, Heidolph Instruments, Schwabach, Germany) in the safety cabinet.

#### Bacterial inoculation into the TIVAP

After the system was set up, LB or TPN priming was performed for all lumens at a speed of 300 μL/min for 1 h. Then, after priming was stopped, ~10^2^ CFUs (per 100 μL) of LF82 or 55989 strains suspended in fresh LB prepared from the overnight culture for in vitro LB experiments or 10^6^ CFUs (per 100 µL) of the 55989pAT881 strain suspended in phosphate-buffered saline (PBS, Corning, no. 21-040-CM, USA) for the in vitro TPN experiment were inoculated into the port using a 1-mL syringe (BD Plastipak) with a Huber needle. The inoculated bacteria were then allowed to adhere to the TIVAP for 3 h without flow.

#### Growth phase

The in vitro experiments consisted of three phases: growth, ALT, and recovery. After 3 h of bacterial inoculation, the continuous flow was started, and non-adherent bacteria were washed out. Biofilm bacteria were grown continuously at a rate of 300 μL/min for 48 h in the LB and 72 h in the TPN experiments, according to a clinical study that revealed a mean time of three days from clinical suspicion of catheter-related infections to administering antibiotics^106^. During the growth phase, the floating cells were sampled at designated time points (1, 4, 24, 48, and 72 h) from the catheter tip under the continuous flow by placing a hemolysis tube for CFU counting and growth frequency analysis on amikacin plates with one time the MIC (described below). The remaining was stored at −80 °C as a glycerol stock.

#### ALT phase (continuous or intermittent antibiotic lock)

After the 48- or 72-h growth phase, the connection tubes upstream and downstream of the TIVAP were aseptically removed from the TIVAP. The connector at the catheter tip was then closed with a plug Luer lock (ARK-PLAS, LNC31-PP0, Fisher Scientific). The remaining fluid in the TIVAP was gently removed from the port using a 1-mL syringe with a Huber needle. Next, 250 μL of amikacin at 8 mg/mL (Sigma; of note, 8 mg/mL corresponds to 500 times the MIC for LF82 and 55989 strains in LB conditions) dissolved in PBS in the ALT_AMK_ group or 250 µL of PBS (in the ALT_PBS_ control group) were slowly injected into the port (Fig. 1a and Supplementary Fig. 1b). The ALT solution was exchanged every 24 h for 10 or 7 days in the continuous ALT regimen or installed for 8 h in the intermittent ALT regimen for seven or four cycles alternating with a 16-h continuous flow. In the intermittent ALT experiments, floating cells were sampled at 1 and 16 h after each ALT cycle.

#### Recovery phase

At the end of (each) ALT phase, the new sterilized connection tubes were connected to the TIVAP upstream and downstream TIVAP to restart the continuous flow for 72 h. In both continuous and intermittent ALT experiments, the floating cells were sampled at designated time points (1, 24, 48, and 72 h) in the recovery phase.

#### Biofilm sample collection from TIVAP at the endpoint

At the endpoint of each experiment, the TIVAP was removed from the system and disinfected externally with 70% ethanol (VWR Chemicals, cat. 83801.290, Radnor, PA, USA) for 15 min. The catheter was aseptically cut every 2-cm long with a sterilized scalpel (hereafter, all preparation was aseptically performed). The silicone septum part was removed from the port and cut into small pieces with a scalpel. For each part, pieces were suspended in one mL LB broth (in a microtube). Biofilm bacteria attaching on/in the port were carefully wiped with a sterile cotton swab (Deltalab, cat. 300201, Barcelona, Spain) moistened with LB broth. The cotton swab tip was then cut at 1 cm and suspended in 1 mL LB broth. All microtubes containing TIVAP parts were vortexed for 1 min at the maximum speed and then sonicated for 10 min in an ultrasonic bath (Branson 5800, Branson Ultrasonics, Danbury, CT, USA). Finally, the biofilm bacterial suspension was used for CFU counting, growth frequency analysis on amikacin plates with one time the MIC, and amikacin MIC testing, and the remaining was stored at −80 °C as a glycerol stock.

#### CFU counting and growth frequency on amikacin plates with one time the MIC

A series of tenfold dilutions was prepared for each sample in 96-well plates, and 10 µL of each dilution (or 100 µL of undiluted samples) was inoculated onto LB agar plates with or without 16 µg/mL amikacin. The plates were incubated at 37°C for 24 h. The growth frequency on amikacin plates at one time the MIC was calculated as follows:

$${Growth\; frequency\; on\; one\; time\; the\; amikacin\; MIC}\,\left( \% \right)=\frac{{{CFU}}_{{amikacin\; at\; one\; time\; the\; MIC}}}{{{CFU}}_{{LB}}}\times 100\,( \% )$$

where CFU_amikacin at one time the MIC_ is the CFU number of each population grown on LB agar plates containing amikacin at 16 µg/mL (one time the MIC), and CFU_LB_ is the one grown on the LB agar plate corresponding to CFU counting.

#### Amikacin susceptibility

The amikacin MIC for the obtained samples was determined using the agar dilution method previously described^43^, with minor modifications. Briefly, the obtained samples were incubated overnight in LB or MH broth from the glycerol stock. The overnight cultures were diluted to ~1.5 × 10^7^ CFU/mL into fresh LB or MH media. Then, the diluted bacterial culture was inoculated onto LB or MH agar plates containing amikacin at 0, 2, 3, 4, 6, 8, 12, 16, 24, 32, 48, 64, 96, 128, 192, and 256 µg/mL using a multipoint replicating device (Boekel, PA, cat. 140500, USA), which transfers ~1 µL of each diluted culture to agar surfaces. The agar plates were incubated at 37 °C for 20 h. The MIC testing was performed with four replicates per sample per agar (two technical replicates prepared from the glycerol stock; this step was repeated twice on different days). The most representative value was adopted as the result.

#### Bioluminescence imaging

In the in vitro TPN experiment using the 55989pAT881 strain, bioluminescence images of biofilm formation on the TIVAP were captured using an imaging system (IVIS Spectrum CT; Perkin Elmer, Waltham, USA) each day during the growth and recovery phases, as well as at 1 and 16 h of the withdrawal period during the ALT phase.

### In vivo TIVAP experiment

#### TIVAP surgery and postoperative care

TIVAP implantation in rats was performed using the same kit as the in vitro experiments, following the previous protocol with modifications^57^. Briefly, after one week of acclimatization, TIVAP implantation surgery was performed for anesthetized 8-week male Crl:CD (SD) rats (Charles River, Wilmington, MA, USA) with isoflurane (ISO-VET, Piramal Critical Care, Bethlehem, PA, USA) on Day 0 as follows: the port part was implanted in the subcutaneous pocket on the back. After the neck skin incision on the ventral side, the catheter was tunneled subcutaneously from the port on the dorsal side to the neck on the ventral side and exposed through the neck skin incision. The catheter was inserted into the right main jugular vein after a partial microincision. The tip of the inserted catheter was advanced to approximately four cm, where the entrance to the right atrium is located. After fixing the catheter in the jugular vein, the surgical sites were closed with sutures. TIVAP patency was attempted to be maintained with a daily heparin lock (250 µL) containing 500 IU/mL on Day 0 and 250 IU/mL thereafter (Sanofi Aventis, Paris, France) in the TIVAP. An anti-inflammatory drug, meloxicam (1.0 mg/kg, Metacam solution injectable 2 mg/mL, Boehringer Ingelheim, Germany), was injected subcutaneously on Days 1–3 to reduce postoperative pain. The incision sites on the back and neck, and the behavior of the rats, were also monitored for 3 days as postoperative care.

#### Bacterial loading into the TIVAP and monitoring of biofilm formations for 3 days

Overnight cultures of *E. coli* 55989pAT881 grown in LB with 50 µg/mL spectinomycin were diluted to 2 × 10^8^ CFU/mL with PBS. Then, after removing the previously installed heparin lock solution from the TIVAP, 50 µL of the diluted culture (1 × 10^7^ CFU as the final bacterial count) was slowly injected into the TIVAP port of rats anesthetized with isoflurane on Day 4 (hereafter, all injection steps were performed on rats under anesthetized conditions). After 24 h of bacterial inoculation, floating bacteria were removed by collecting the solution from the port. Then, 250 µL of TPN infusion was installed for 3 days (Days 5–8) into the TIVAP of isoflurane anesthetized rats and exchanged daily by collecting the previous solution from the TIVAP and injecting a new solution. If the previous infusion could not be collected due to reduced catheter patency, a new solution was gently injected without the need for a collection procedure. Ceftriaxone (200 mg/kg, Sigma, C5793) was also administered subcutaneously once daily until the end of the experiment (Day 15) to prevent systemic infections. Bioluminescence images of biofilm formation on the TIVAP were regularly captured on the dorsal and ventral sides of isoflurane anesthetized rats using IVIS Spectrum CT, including the condition before bacterial loading.

#### Intermittent ALT and recovery phases

After the 3-day biofilm formation, 250 µL of amikacin lock solutions at 8 mg/mL dissolved in PBS containing heparin (250 IU/mL of the final conc.) was installed into the TIVAP of isoflurane anesthetized rats for eight hours. About 250 µL of PBS with heparin at 250 IU/mL was used in the ALT_PBS_ control group. Then, 250 µL of the TPN infusion was installed into the TIVAP for 16 h. This intermittent treatment was repeated for four cycles on Days 8–12. Finally, the TPN infusion was installed into the TIVAP for 3 days (Days 12–15), and the solution was exchanged daily. Bioluminescence images of biofilm formation on the TIVAP of isoflurane anesthetized rats were captured before and after intermittent ALT procedures and each day in the recovery phase. Note that during the biofilm growth, regrowth, and recovery periods, TPN was administered as a 250 µL instillation, not as a continuous flow, due to the impossibility of immobilizing the rat.

#### TIVAP extraction from euthanized rats

On Day 15, after the rats were euthanized with carbon dioxide, the TIVAP was aseptically removed from the euthanized rat. Then, the bioluminescence images of the biofilms in the extracted TIVAP were captured. Next, after the surface of the TIVAP was carefully disinfected with 70% ethanol, the TIVAP was disassembled into silicone septum, port, and catheter parts as described above. Biofilm bacteria on each part were analyzed for CFU counting, growth frequency analysis on amikacin plates at 1 time the MIC, and amikacin MIC testing.

### Whole genome sequencing, assembly, and data analyses

Whole-population genome sequencing (WGS) was applied to endpoint biofilm populations treated with continuous or intermittent amikacin in the in vitro and in vivo experiments to investigate gene mutations. LF82 biofilm samples saved in 15% glycerol (obtained from the continuous and intermittent in vitro LB experiments) were directly transferred from the glycerol stock to fresh LB broth and regrown overnight at 37 °C under shaking conditions. Meanwhile, for each sample from 55989 biofilm samples obtained from the in vitro LB experiment and 55989pAT881 from the in vitro TPN experiments, given the relatively low frequency of mutants growing on amikacin plates at one time the MIC in the growth frequency analysis and considering that the corresponding mutations would likely be missed by WGS analysis since Breseq has a detection limit of 5%, colonies growing on amikacin plates at one time the MIC were pooled and incubated in LB broth without antibiotics overnight at 37 °C under shaking conditions. This enriched method was also applied to 55989pAT881 biofilm samples from the in vivo experiment. Similarly to the previous study^26^, the genomic DNA was then extracted from these cultures, prepared for whole genome sequencing using the Nextera XT DNA library preparation kit (Illumina, San Diego, CA, USA), and sequenced on the Hiseq and Miseq platforms (Illumina) with an average depth of 150 ×. Read quality was assessed using FastQC version 0.11.9 (http://www.bioinformatics.babraham.ac.uk/projects/fastqc/) and trimmed using Trimmomatic version 0.39^107^. The trimmed sequence reads were analyzed by BreSeq version 0.35.0^108^ to detect genetic variants.

### Statistical analysis

Data were presented as the mean ± standard error (SE), with or without individual data points, from three independent biological experiments. Statistical analyses were conducted using Prism 10.2.3 (GraphPad Software Inc.). They corresponded to an unpaired two-tailed *t*-test with Welch’s correction between two groups and to the Welch one-way analysis of variance (ANOVA) with Dunnett post hoc test for comparisons involving more than two groups. *P* values <0.05 were deemed statistically significant.

### Ethics approval

The French Ministry of Agriculture accredited the animal facility to perform experiments on live rodents (accreditation number A75-15 01, issued on 20 October 2027, and number A75-15 02, issued on 20 October 2027) in compliance with French and European regulations on the care and protection of laboratory animals (EC Directive 2010/63, French Law 2013-118, issued on 1 February 2013). All experiments were approved by the Ethics Committee #89 (le CETEA, Institut Pasteur) and registered under the reference APAFIS #45216-2023101919069048 v1).

## Supplementary information

Supplementary material

## Data Availability

Supplementary Data 1 corresponds to the Mutations identified using Breseq in end-point biofilm populations at a frequency higher than 5%. It can be downloaded at https://zenodo.org/records/17655296. Supplementary Data 2 file corresponds to the raw data of the different experiments presented in this manuscript. It can be downloaded at https://zenodo.org/records/17655323.
