# Supplementary material for: Intermittent antibiotic exposure of Escherichia coli biofilms drives resistance in catheter-associated infection models
Source: NPJ Biofilms Microbiomes. 2026 Jan 17;12:41. doi: 10.1038/s41522-025-00906-4 (PMC12894844; doi:10.1038/s41522-025-00906-4)
Supplement: Supplementary file 1 — Supplementary material [file 41522_2025_906_MOESM1_ESM.pdf]

Supplementary information

Intermittent Antibiotic Exposure of *Escherichia coli* Biofilms Drives Resistance in Catheter-Associated Infection Models

Yutaka YOSHII, Stanislas THIRIET-RUPERT, David LEBEAUX, Jean-Marc GHIGO, Christophe BELOIN

Supplementary information includes:

Supplementary Data 1: Mutations identified using Breseq in end-point biofilm populations at frequencies ≥5%.
It can be downloaded at https://zenodo.org/records/17655296.

Supplementary Data 2: contains the raw data of the different experiments.
It can be downloaded at https://zenodo.org/records/17655323..

Supplementary Table 1

Supplementary Figures 1 to 5

Supplementary Table 1. Bacterial strains and plasmids used in this study

E. coli strains	Description	Reference
LF82 (LF82Δ*ampC*::mars)	A derivative of adherent invasive *E. coli* LF82[1)] isolated from an ileal biopsy of a Crohn's disease patient. LF82Δ*ampC*::mars-FRT. Deletion of *ampC* by mars-KmFRT and removal of the kanamycin resistance.	2)
55989	Enteroaggregative *E. coli*	3)
55989pAT881	A 55989 strain transformed with stable plasmid pAT881 providing production of bioluminescence	4)

Supplementary References

1) Glasser AL, Boudeau J, Barnich N, Perruchot MH, Colombel JF, Darfeuille-Michaud A. Adherent invasive *Escherichia coli* strains from patients with Crohn's disease survive and replicate within macrophages without inducing host cell death. *Infect Immun*. 2001; 69:5529-37. DOI: 10.1128/IAI.69.9.5529-5537.2001
2) Usui M, Yoshii Y, Thiriet-Rupert S, Ghigo J-M, Beloin C. Intermittent antibiotic treatment of bacterial biofilms favors the rapid evolution of resistance. *Communications Biology* 2023; 6:275. DOI: 10.1038/s42003-023-04601-y
3) Bernier C, Gounon P, Le Bouguénec C. Identification of an aggregative adhesion fimbria (AAF) type III-encoding operon in enteroaggregative *Escherichia coli* as a sensitive probe for detecting the AAF-encoding operon family. *Infect Immun.* 2002l; 70:4302-11. DOI: 10.1128/IAI.70.8.4302-4311.2002
4) Chauhan A, Ghigo J-M, Beloin C. Study of in vivo catheter biofilm infections using pediatric central venous catheter implanted in rat. *Nature Protocols*. 2016;11(3):525-541. DOI: 10.1038/nprot.2016.033

Supplementary Figures

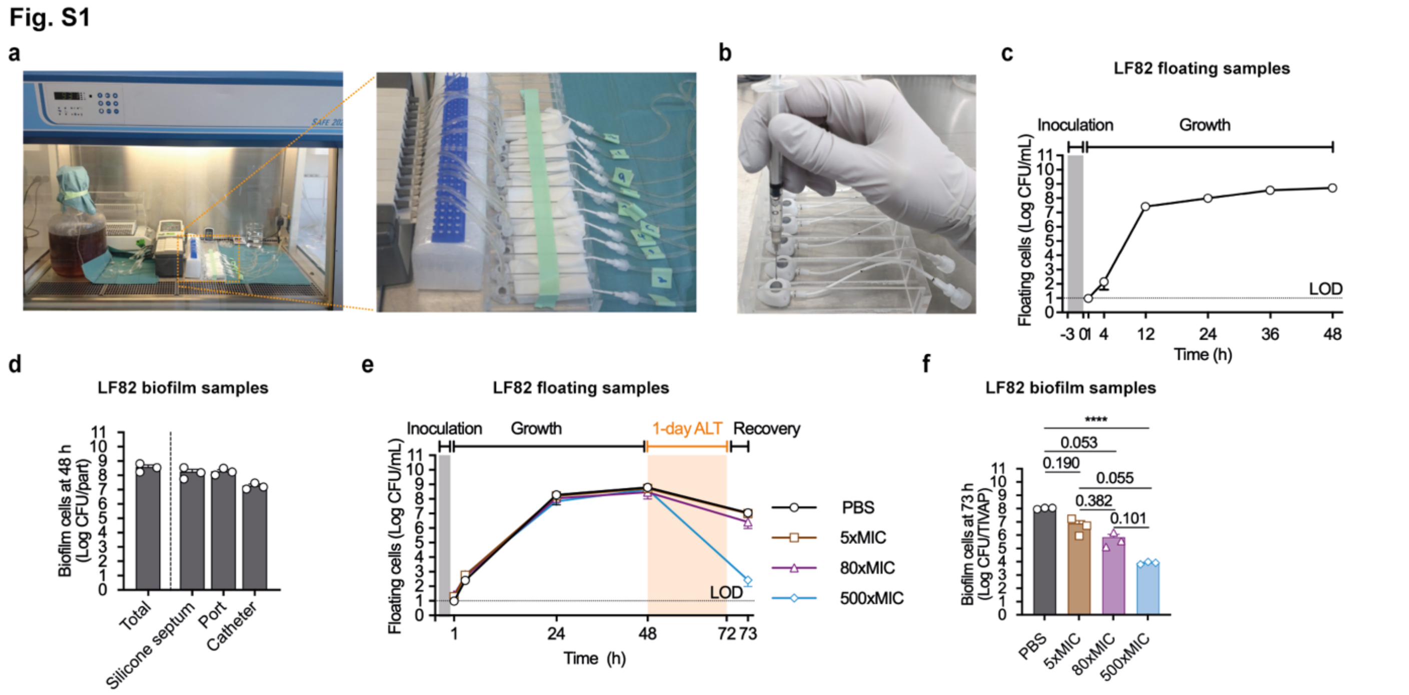

Supplementary Figure 1. LF82 biofilm formation on *in vitro* TIVAP under continuous LB flow. (**a**) Representative photographs showing the *in vitro* TIVAP model combined with continuous LB flow. (**b**) The actual lock therapy step after removing the continuous flow system. (**c**) Time course of CFUs of floating cell samples collected from the tips of the TIVAP catheters after 48-hour continuous LB flow incubation. (**d**) CFUs of biofilm samples formed on each TIVAP part (silicone septum, port, and catheter) after 48-hour continuous LB flow in the same experiment with (c). (**e**) Time course of CFUs of floating cell samples collected from TIVAPs treated with a 24-hour amikacin lock at different concentrations (PBS control, 5 times, 80 times, and 500 times MIC), followed by 1-hour recovery continuous LB flow. (**f**) CFUs of biofilm samples at the end-point in the same experiment with (c). Three TIVAPs were used for each condition in each experiment. LOD indicates the lowest CFU that can be measured. Means, individual values, and standard errors from three biological replicates (TIVAPs) are shown (Welch ANOVA with Dunnett post hoc test, ****p <0.0001).

Fig. S2

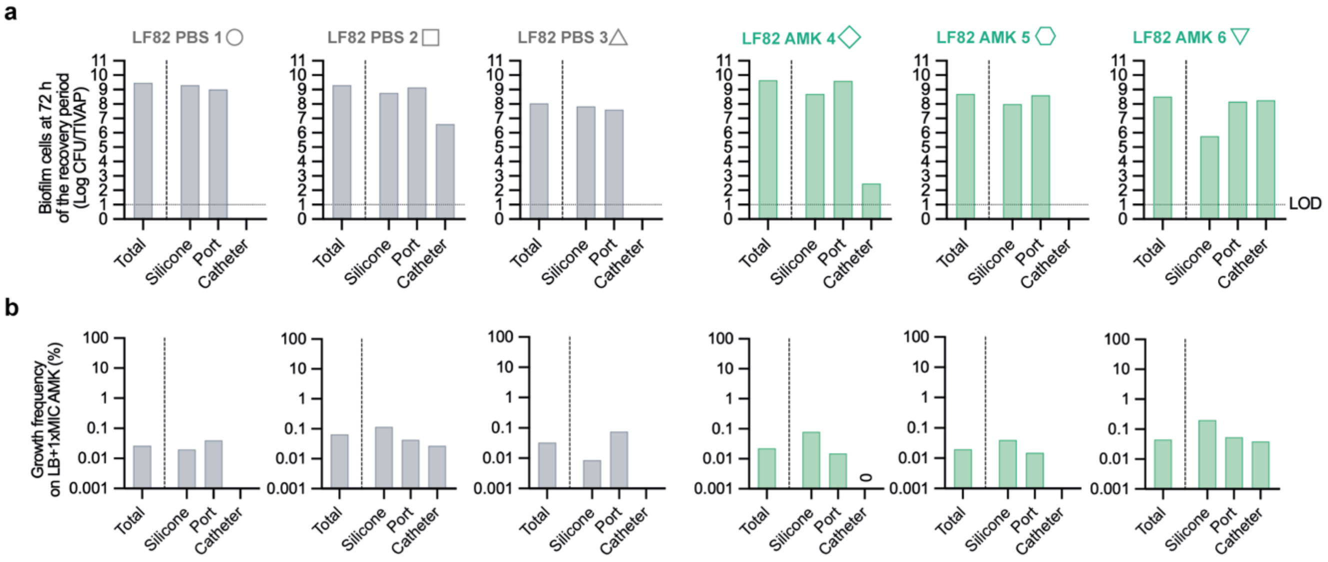

c

	PBS 1 ○		PBS 2 □		PBS 3 △	
	LB	MH	LB	MH	LB	MH
Slicone	16	4	16	4	16	4
Port	16	4	16	4	16	4
Catheter	-	-	16	4	-	-

	AMK 4 ◇		AMK 5 ⬡		AMK 6 ▽	
	LB	MH	LB	MH	LB	MH
Slicone	16	4	16	4	16	4
Port	16	4	16	4	16	4
Catheter	16	4	-	-	16	4

Supplementary Figure 2. Individual TIVAP sample data in the 7-day continuous *in vitro* experiment. (**a**) CFUs of biofilm samples of each TIVAP part in individual TIVAPs. Using the total CFU of individual TIVAPs, Figure 1f was generated. (**b**) Growth frequencies of biofilm cell samples of each TIVAP part on amikacin plates at 1 time the MIC. Total growth frequencies are also shown as part of Figure 1g. (**c**) Amikacin MIC for biofilm population samples obtained from each TIVAP part of individual TIVAPs on LB and Mueller-Hinton (MH) media.

Fig. S3

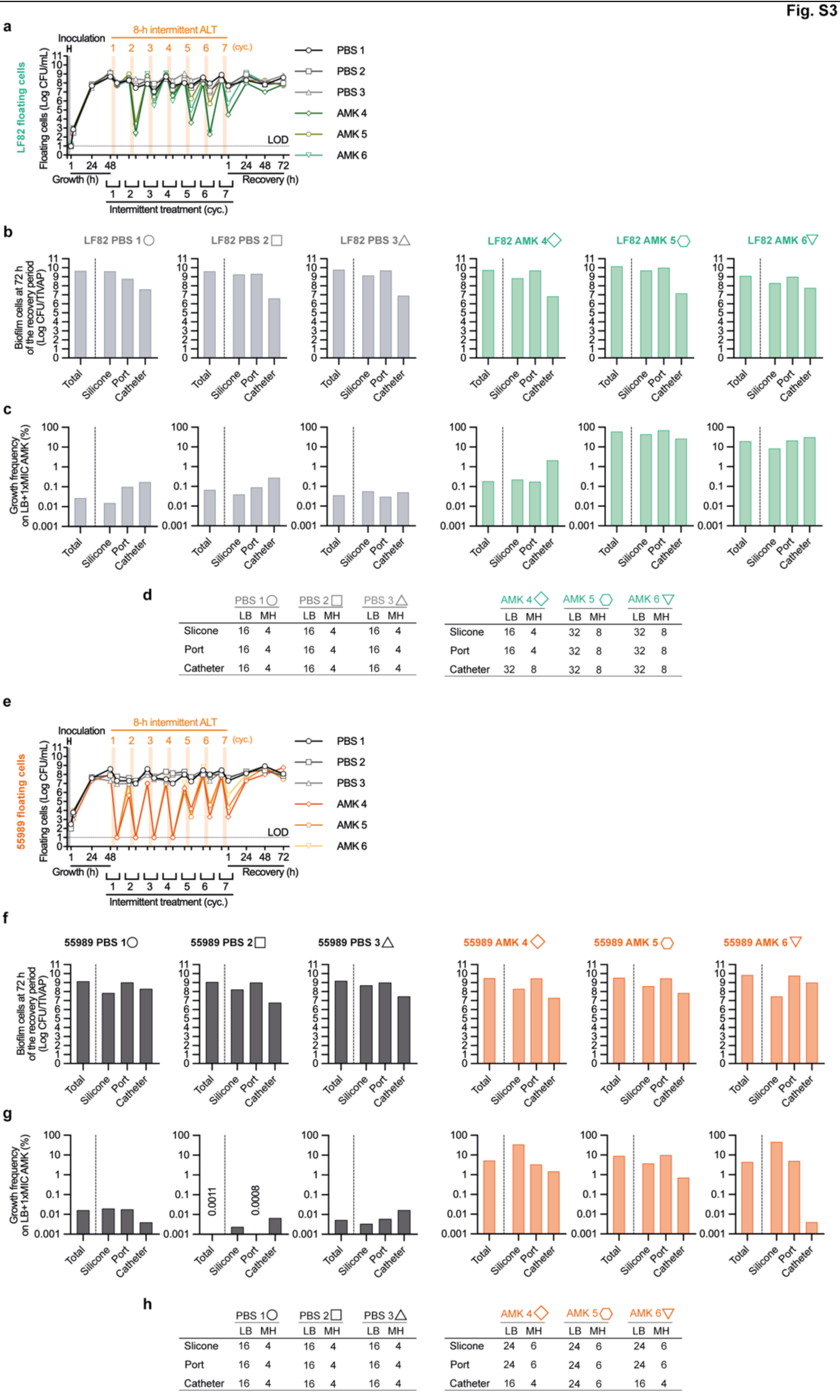

d

	PBS 1 LB	PBS 1 MH	PBS 2 LB	PBS 2 MH	PBS 3 LB	PBS 3 MH
Slicone	16	4	16	4	16	4
Port	16	4	16	4	16	4
Catheter	16	4	16	4	16	4

	AMK 4 LB	AMK 4 MH	AMK 5 LB	AMK 5 MH	AMK 6 LB	AMK 6 MH
Slicone	16	4	32	8	32	8
Port	16	4	32	8	32	8
Catheter	32	8	32	8	32	8

h

	PBS 1 LB	PBS 1 MH	PBS 2 LB	PBS 2 MH	PBS 3 LB	PBS 3 MH
Slicone	16	4	16	4	16	4
Port	16	4	16	4	16	4
Catheter	16	4	16	4	16	4

	AMK 4 LB	AMK 4 MH	AMK 5 LB	AMK 5 MH	AMK 6 LB	AMK 6 MH
Slicone	24	6	24	6	24	6
Port	24	6	24	6	24	6
Catheter	16	4	24	6	16	4

Supplementary Figure 3. Individual TIVAP sample data in the *in vitro* seven-cycle intermittent amikacin lock experiment. The data encompasses the following key parameters: CFUs of biofilm samples, growth frequency, and amikacin MIC in the seven cycles of the intermittent amikacin lock experiment with LB medium. (**a–d**) Individual LF82 TIVAP sample data: CFU time course (a), endpoint biofilm CFU (b), growth frequencies on amikacin plates with 1 time the MIC (c), and population amikacin MIC for biofilm samples (d). (**e–h**) Individual 55989 TIVAP sample data: CFU time course (e), endpoint biofilm CFU (f), growth frequencies on amikacin plates with 1 time the MIC (g), and population amikacin MIC for biofilm samples (h). Using the data in (a, e), Figure 2b and c were generated. Using total CFUs of

individual TIVAPs in (b, f), Figure 2d and e were generated. Total growth frequencies in (c, g) are shown as part of Figure 2f and g. The highest MIC values in each condition (d, h) are shown in Figure 2h and i.

Fig. S4

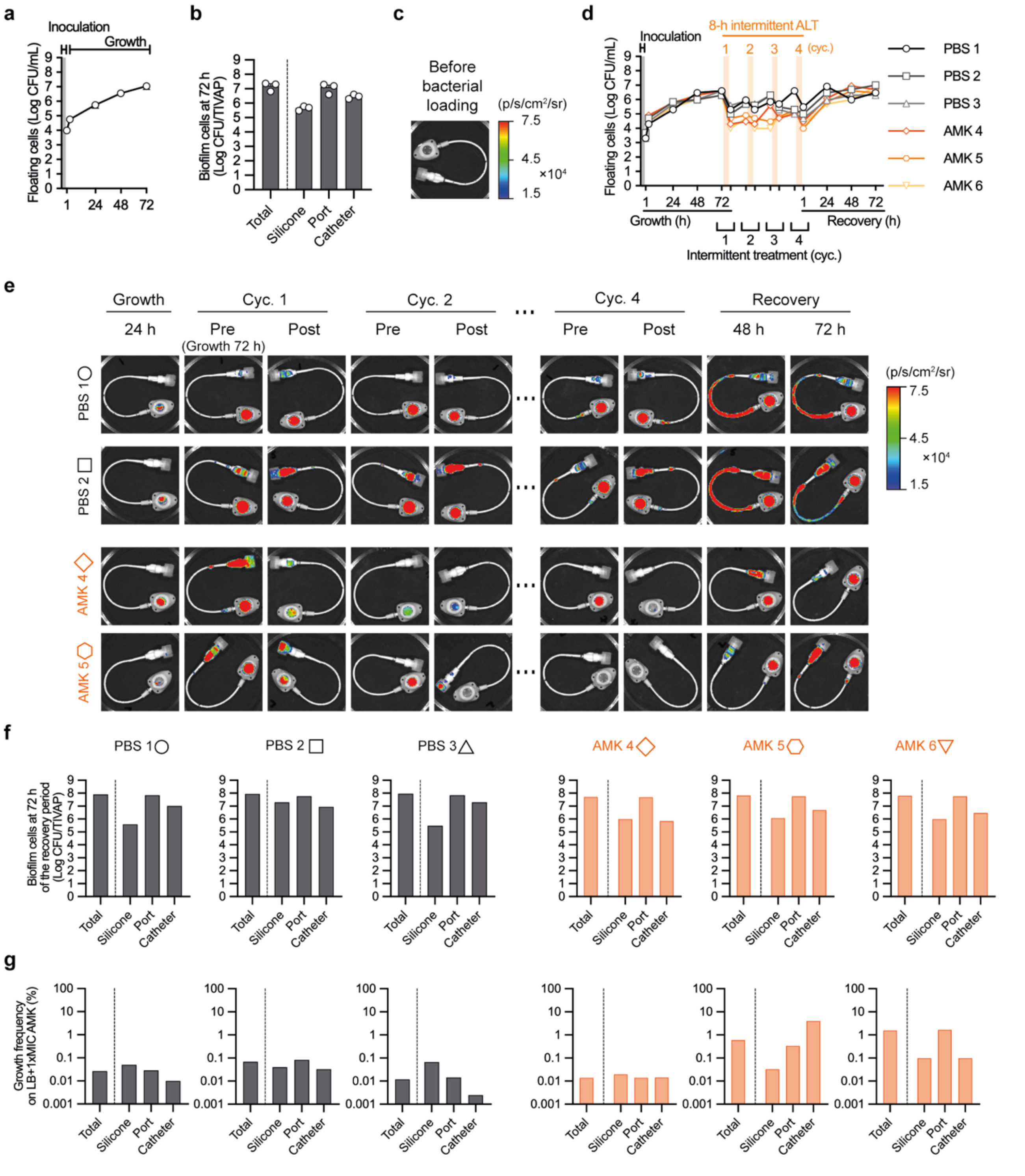

h

	PBS 1○ LB	PBS 1○ MH	PBS 2□ LB	PBS 2□ MH	PBS 3△ LB	PBS 3△ MH
Slicone	16	4	16	4	16	4
Port	16	4	16	4	16	4
Catheter	16	4	16	4	16	4

	AMK 4◇ LB	AMK 4◇ MH	AMK 5⬡ LB	AMK 5⬡ MH	AMK 6▽ LB	AMK 6▽ MH
Slicone	16	4	16	4	16	4
Port	16	4	24	6	24	6
Catheter	16	4	24	6	16	4

Supplementary Figure 4. *In vitro* TIVAP biofilms formed by bioluminescent 55989pAT881 under continuous TPN flow and individual TIVAP sample data for four cycles of intermittent amikacin lock. (**a**) Time course of CFUs of floating cell samples obtained from the catheter tips of TIVAPs after 72-hour continuous TPN flow. (**b**) CFUs of endpoint biofilm samples formed on each TIVAP part (silicone septum, port, and catheter) in the same experiment as (a). (**c**) Bioluminescence image of TIVAP before bacterial loading. (**d**) Individual data for the time course

of floating cell samples obtained from each TIVAP. Using the data in (d), Figure 3b was generated. (**e**) Time course bioluminescence images of biofilms formed on TIVAPs. (**f**) CFUs of biofilm samples of each TIVAP part. Using the total CFUs of each TIVAP, Figure 3c was generated. (**g**) Growth frequencies of biofilm samples of each TIVAP part on amikacin plates at 1 time the MIC. Total growth frequencies are shown as part of Figure 3e. (**h**) Amikacin MIC for biofilm population samples obtained from each TIVAP part (silicone septum, port, and catheter) on LB and MH media. The highest MIC values in each condition in (h) are listed in Figure 3f.

Fig. S5

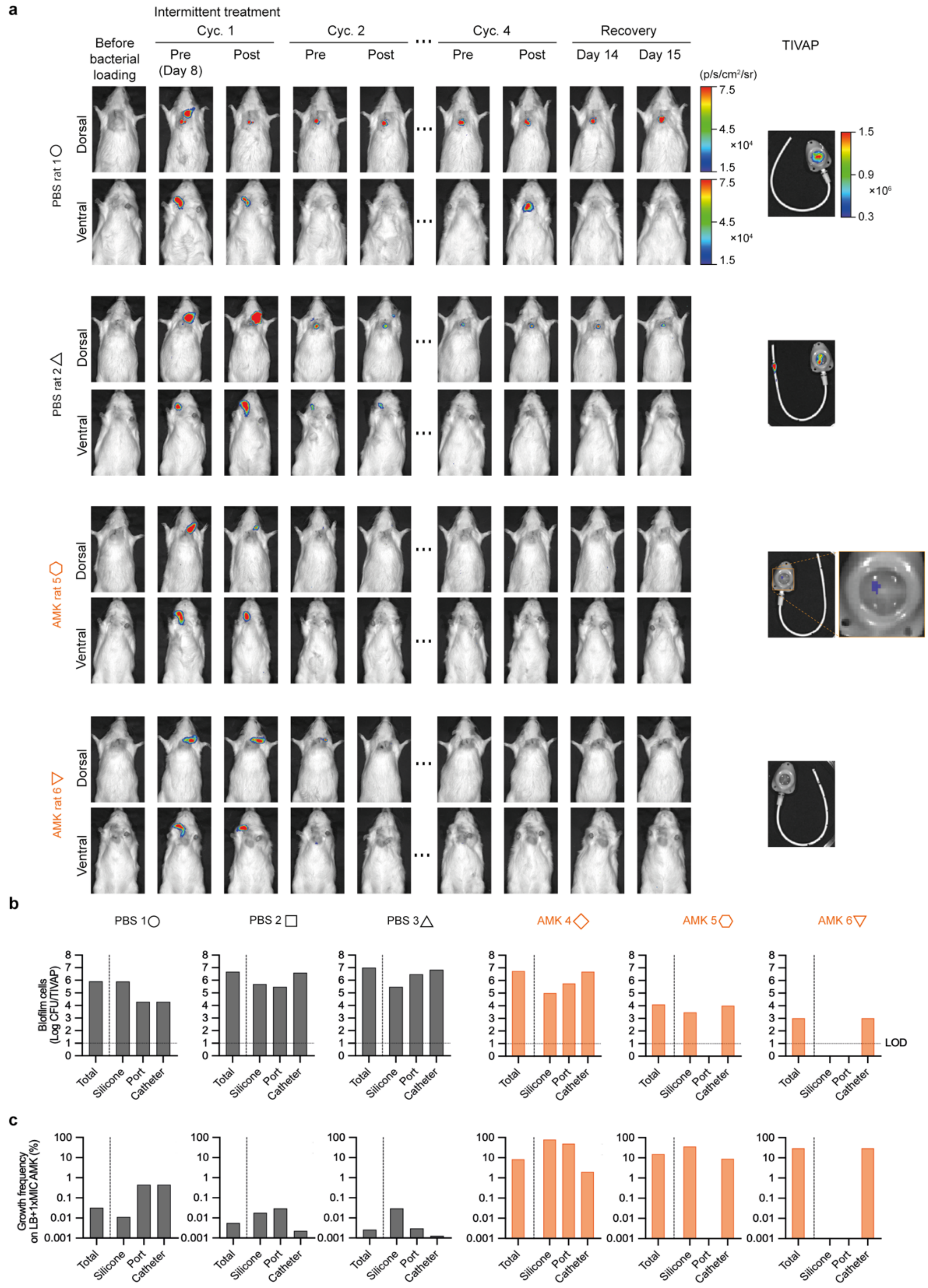

d

	PBS 1○		PBS 2□		PBS 3△	
	LB	MH	LB	MH	LB	MH
Slicone	16	4	16	4	16	4
Port	16	4	16	4	16	4
Catheter	16	4	16	4	16	4

	AMK 4◇		AMK 5⬡		AMK 6▽	
	LB	MH	LB	MH	LB	MH
Slicone	24	6	24	6	-	-
Port	24	6	-	-	-	-
Catheter	24	6	24	6	96	24

Supplementary Figure 5. Individual *in vivo* TIVAP sample data. The following are presented: the time course of bioluminescence images, CFUs of biofilm samples, growth frequency and amikacin MIC. (**a**) Individual time course of bioluminescence images of 55989pAT881 biofilms and TIVAPs at the endpoint treated with or without amikacin lock. (**b**) CFUs of biofilm samples of each TIVAP part in individual TIVAPs. Using the total CFUs of each TIVAP, Figure 4d was generated. (**c**) Growth frequencies of biofilm samples of each TIVAP part on amikacin plates at 1 time the MIC. Using the total growth frequencies of each TIVAP, Figure 4e was generated. (**d**) Population amikacin MIC for biofilm samples obtained from each TIVAP part (silicone septum, port, and catheter) on LB and MH media. The highest and most frequent MIC values in each condition in (d) were listed in Figure 4f.
